# Recent advances in field cancerization and management of multiple cutaneous squamous cell carcinomas

**DOI:** 10.12688/f1000research.12837.1

**Published:** 2018-06-01

**Authors:** Sean R. Christensen

**Affiliations:** 1 Section of Dermatologic Surgery and Cutaneous Oncology, Department of Dermatology, Yale University, New Haven, CT, USA

**Keywords:** cutaneous squamous cell carcinoma, field cancerization, lesion-directed therapy

## Abstract

Cutaneous squamous cell carcinoma (SCC) is among the most common cancers in humans, and many patients with SCC will develop multiple tumors within their lifetime. The field cancerization concept, originally proposed over 60 years ago, hypothesized that multiple primary cancers may arise simultaneously and coexist with subclinical precursor lesions within a defined field. Genetic sequencing of SCC and precursor lesions has identified what may be the earliest clonal proliferations in SCC development and confirmed that field cancerization in the skin is mediated by ultraviolet radiation. For patients with multiple SCCs and severe actinic damage, treatment of precursor lesions within a cancerized field can decrease the risk of subsequent cancer development. Sunblock is an effective intervention for field cancerization, even in patients with established disease. There is now direct evidence that field therapy with topical 5-fluorouracil is effective in reducing the incidence of subsequent SCC, and there is indirect evidence suggesting that topical imiquimod, topical ingenol mebutate, and photodynamic therapy are similarly effective. There is limited direct evidence to show that systemic acitretin or nicotinamide can decrease incident SCC in patients with field cancerization. In this review, an approach to the management of patients with multiple SCCs and field cancerization is presented along with the rationale to support field-directed therapy.

## Introduction

Cutaneous squamous cell carcinoma (SCC) is the second most common malignancy in humans; up to 1 million SCC tumors are treated annually in the United States ^1^. Over half of patients with diagnosed SCC will develop multiple tumors within their lifetime. Although the majority of SCCs of the skin are localized tumors with an exceptionally good prognosis, approximately 5% of SCCs recur locally after definitive treatment, 2–3% exhibit nodal metastasis, and 1–2% are fatal ^2, 3^. In addition to causing between 4,000 and 9,000 deaths annually, SCC and its associated skin lesions have an enormous financial burden at over $4 billion per year in the United States ^4^.

**Figure 1.**
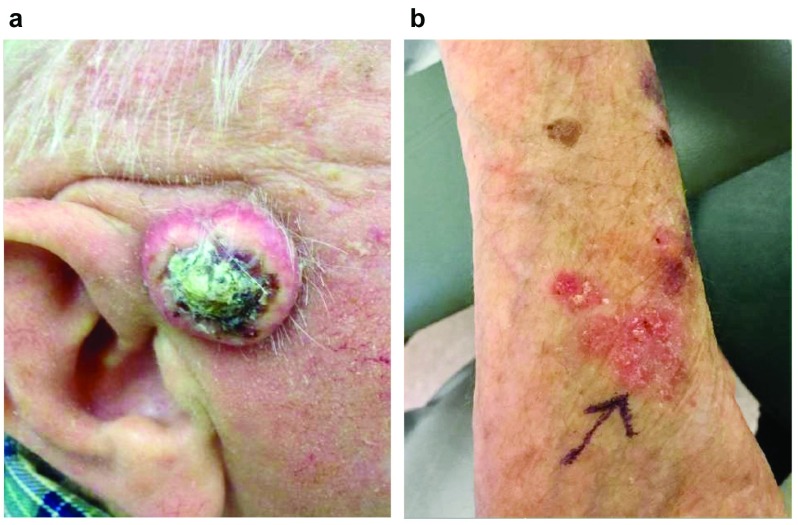
Clinical presentation of squamous cell carcinoma (SCC) and SCC *in situ*. ( **a**) Invasive SCC presenting as a firm ulcerated lesion with central core of keratinaceous and hemorrhagic debris on the preauricular cheek. ( **b**) SCC *in situ* presenting as multi-focal and poorly demarcated pink scaly plaques on the dorsal forearm.

**Figure 2.**
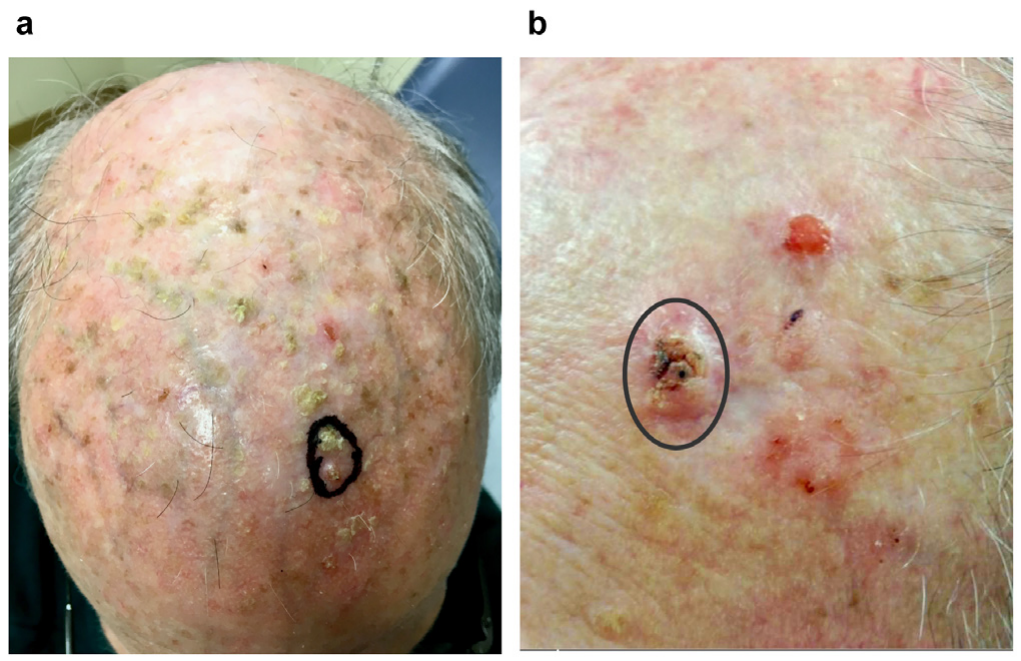
Clinical presentation of field cancerization. ( **a**) Squamous cell carcinoma (SCC) *in situ* (circled lesion) presenting on the scalp with chronic actinic damage and innumerable small, gritty actinic keratoses. It is not clear which lesions are precursors and which may have progressed to SCC. ( **b**) SCC (circled lesion) presenting on the forehead in close proximity to three additional lesions. Two of the satellite lesions were SCC *in situ* that was discontiguous with the primary SCC, suggesting simultaneous but independent development of the lesions.

Cutaneous SCC typically presents in older patients on chronically sun-exposed skin as a scaly erythematous papule that may have ulceration and hemorrhagic crust. Some lesions have more pronounced hyperkeratosis, and larger lesions may exhibit a central core of hard keratin and hemorrhagic debris ( Figure 1a). Lesions may bleed or be tender to palpation but often are asymptomatic. SCC *in situ* is an intra-epidermal malignancy (not breaching the basement membrane) that often presents as a pink scaly plaque ( Figure 1b) and has a low but reported risk of progression to invasive SCC ^5^. Although the classic presentation of SCC is a solitary lesion, some patients present with multiple lesions that may be admixed with premalignant lesions known as actinic keratoses ( Figure 2). In these patients, lesion-directed therapy of dozens of individual neoplasms does not adequately address their underlying pathology, which is best described as field cancerization. This review will focus on recent developments in the pathogenesis and management of patients with multiple cutaneous SCCs and field cancerization.

## Lesion-directed therapy of squamous cell carcinoma

Cutaneous SCC is most commonly treated with lesion-directed therapy using surgical excision or destruction. Standard excision with an appropriate margin of clinically normal skin (typically 4–6 mm) or destruction with electrodesiccation and curettage results in cure rates as high as 90–95% for typical SCC ^6, 7^. SCC with significant subclinical extension, or local invasion of the tumor beyond grossly visible margins, is best treated with Mohs micrographic surgery, a specialized method of staged excision in which the entire deep and peripheral surgical margin is microscopically examined by the surgeon with rapid intra-operative pathology ^8^. Established risk factors for subclinical extension and aggressive behavior of SCC include recurrent tumors, tumor diameter greater than 2 cm, depth of invasion greater than 6 mm, poorly differentiated or desmoplastic histologic features, perineural invasion, immunosuppression, and tumor location on the scalp, ears, eyelid, nose, or lip ^9– 11^. The superiority of Mohs micrographic surgery over standard excision for these high-risk tumors has been documented in retrospective studies ^12^, and the Mohs technique is currently recommended for SCC with high-risk features or in anatomically sensitive locations ^13^.

For SCC not amenable to surgery, non-surgical treatments may be used, albeit with lower cure rates. Topical medical therapy of individual SCC *in situ* lesions with either imiquimod or 5-fluorouracil (5FU) has been reported with cure rates of 73–86% and 48–69%, respectively ^14– 17^, but neither of these treatments is consistently effective for invasive SCC. Similarly, photodynamic therapy (PDT) with either aminolevulinic acid or methyl aminolevulinate may achieve clinical cure rates of 76–82% for SCC *in situ* ^16– 18^. Lesion-directed cryotherapy with liquid nitrogen (a form of physical destruction of the lesion) is commonly used for low-risk SCC *in situ* and precursor lesions of actinic keratosis (AK). Although cryotherapy is highly operator-dependent, one trial reported a complete response rate for SCC *in situ* of 67% at 1 year, which was comparable to 5FU and inferior to PDT ^17^. Intra-lesional chemotherapy (direct local injection into the tumor) with agents such as 5FU, methotrexate, bleomycin, or type I interferon has successfully treated SCC in several published case series, often specifically addressing the keratoacanthoma type of SCC that is characterized by rapid growth and occasional spontaneous involution ^19^. Because intra-lesional chemotherapy has not been studied in controlled clinical trials, its use is often reserved for patients who are poor surgical candidates or who have such a high burden of individual lesions that surgery is not practical. Although medical treatment of SCC has decreased efficacy for individual lesions relative to surgery, several of these modalities have the benefit of potentially treating a broad field around the index lesion, which may inhibit the development of subsequent SCC. Patients with multiple SCCs often benefit from a combination of medical and surgical field-directed and lesion-directed treatment approaches as discussed below.

Radiation therapy has long been employed in the treatment of SCC and may be preferable to surgical treatment in specific circumstances. Low-risk SCC treated with superficial X-ray therapy has a reported 5-year recurrence rate of 5.8% in one series ^20^. Treatment of larger SCCs, poorly differentiated tumors, or tumors on the scalp, ears, or lips with superficial radiation may result in increased recurrence rates of up to 30% ^21^. More intense radiation therapy with electron beam or megavoltage photons has been reported as monotherapy for advanced SCC, but high-quality outcome data are lacking ^22^. In addition, because of the risk of secondary malignancy decades after radiation therapy, this treatment is relatively contraindicated in younger patients with a long life expectancy. Finally, advanced SCC with lymph node or visceral metastasis is best managed with multi-modal therapy, including surgery, radiation, and potentially systemic chemotherapy. More recently, immunotherapy with monoclonal antibodies to the immune checkpoint receptor programmed death 1 (PD-1) on T cells has been reported as a successful treatment to induce T cell-mediated rejection of advanced SCC that is unresponsive to standard therapy ^23, 24^. Whether this therapy can be adapted for patients with multiple primary SCCs remains to be determined.

## Field cancerization

In 1953, Slaughter and colleagues described the concept of field cancerization on the basis of their study of pathologic changes in the epithelium surrounding oropharyngeal SCC ^25^. In that seminal report, the authors observed that all cases of oropharyngeal SCC had pathologic dysplasia and foci of SCC *in situ* in the adjacent mucosa that was grossly normal (no clinical evidence of disease). Importantly, many of these changes were not contiguous with the primary tumor and represented separate islands of dysplastic epithelium. In addition, 11% of oropharyngeal SCCs presented with two separate primary lesions, a rate 10-fold greater than expected on the basis of disease prevalence. Together, these findings support the independent or co-incident development of multiple lesions rather than the linear progression of a single mutant clone. The paradigm of field cancerization was thus proposed on the basis of the hypothesis that an area, or field, of epithelium is altered by a regional carcinogenic activity. This regional injury, which may be acute or chronic, causes irreversible genetic changes in multiple clonal populations of cells, one or several of which eventually manifest in cancer.

Although this concept of field cancerization has long been observed in the skin, Slaughter’s original pathologic findings were recently recapitulated for cutaneous SCC. In excision specimens of cutaneous SCC, 57% had separate (discontiguous) foci of dysplasia that were not detected clinically but were pathologically diagnostic of AK ^26^. In addition, both histopathologic biopsies and non-invasive imaging with optical coherence tomography of cutaneous regions of suspected field cancerization confirmed that 79% of grossly normal skin samples in these fields had evidence of dysplasia or occult carcinoma ^27^. The field cancer paradigm has two important implications for the management of cutaneous SCC. First, because SCC arises from multi-focal areas of precancerous change, the presence of at least one SCC confers an increased risk of subsequent SCC. It has been shown that after a single SCC, there is a 42% risk of subsequent SCC within 5 years and this risk increases to 72% in patients with two or more SCCs ^28^. Second, clinical recurrence of SCC after complete surgical excision may in fact represent the development of a new primary cancer, and recurrence risk may correlate with the degree of field cancerization. Indeed, it was found that patients with two to nine SCCs exhibit a twofold increased risk of recurrence compared with patients with a single SCC, and patients with a lifetime history of 10 or more SCCs exhibit a 12-fold increased risk of local recurrence as well as an 11-fold increased risk of nodal metastasis ^29^. In these patients with a heavy burden of individual cancers, understanding the mechanism of field cancerization and SCC evolution from precursor lesions has spurred development of therapeutic strategies to prevent or suppress the development of subsequent SCC.

## Precursor lesions and the mechanistic basis of field cancer

The primary driver of epidermal carcinogenesis is solar ultraviolet (UV) radiation. Epidemiologic studies have demonstrated a 10-fold increase in SCC incidence with increasing ambient solar radiation ^30^. From a mechanistic standpoint, UV radiation directly (primarily UVB wavelengths, 290–320 nm) and indirectly (primarily UVA wavelengths, 320–400 nm) damages epidermal DNA, leading to somatic mutations in genes that confer a growth advantage and facilitate malignant transformation ^31^. Somatic mutations, both inactivating mutations in tumor suppressor genes and activating mutations in oncogenes, accumulate over time in a sequential fashion. According to the model of multi-step carcinogenesis, increasing somatic mutations induce clonal proliferations that progress from microscopic precursors to grossly visible lesions during the development of carcinoma ^32^. A second mechanistic effect of UV radiation is local immunosuppression that facilitates epidermal carcinogenesis. Studies in human volunteers have defined both acute and chronic effects of UVB in inhibiting adaptive immune responses to contact allergens applied to the skin and shown that this UV-mediated immunosuppression correlates with SCC risk ^33^. Given the established association of therapeutic immunosuppression (for example, in organ transplant recipients) with dramatically increased SCC risk, it is clear that effective anti-tumor immunity plays an important role in limiting the progression of precursor lesions in the skin.

The most characteristic precursor lesion in the skin is AK, a small papule with gritty scale that has pathologic features of epidermal dysplasia without overt carcinoma. Like SCCs, AKs are causally associated with chronic UV exposure. Although the risk of an individual AK progressing to carcinoma is low, the presence and number of AKs are correlated with SCC risk ^34, 35^. AK is a clear clinical indicator of field cancer, and some patients may present with dozens of lesions in a single anatomic field, conferring a high risk of subsequent SCC ( Figure 2a).

SCC and its precursor lesions have similar genetic driver mutations, providing further support for the field cancer model. The most commonly described somatic mutations in cutaneous SCC are loss-of-function mutations in the tumor suppressors *TP53* and *NOTCH1*, identified in up to 95% and 75% of SCCs, respectively ^36– 38^. Notably, over three-quarters of somatic mutations in SCC are UV-signature mutations induced by direct interaction of UVB with pyrimidine bases in DNA. Approximately two-thirds of AKs harbor mutations in *TP53* and these mutations have also been identified in 14% of epidermal cells in sun-exposed but normal skin ^39, 40^. Cells with mutated *TP53* proliferate in the epidermis as a clonal expansion or “patch” under continuing UV exposure, and animal studies have confirmed that UVB promotes clone expansion but that stopping UVB exposure causes some clones to regress ^41, 42^. Similarly, *NOTCH1* mutations are present in clinically and histologically normal skin adjacent to SCC and appear to arise by contiguous growth of a clonal precursor ^38^.

**Figure 3.**
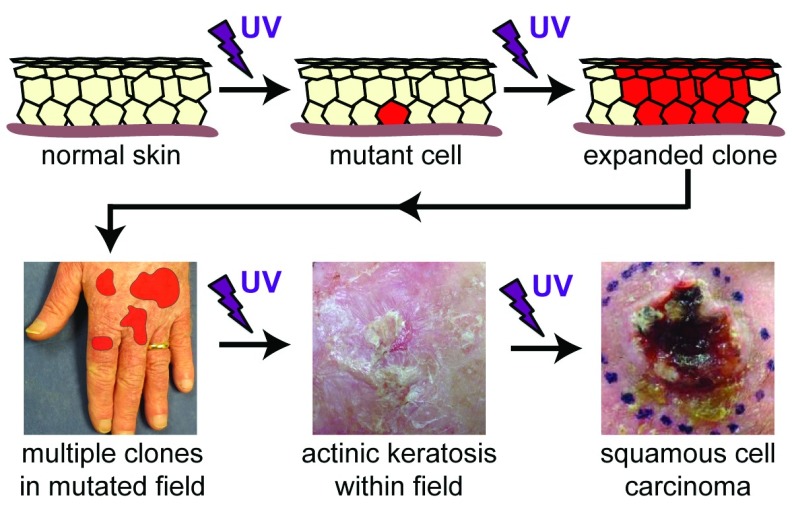
Pathogenesis of field cancerization. Ultraviolet (UV) radiation directly induces mutation in a single keratinocyte. If this mutation provides a selective growth advantage, such as with loss of functional *TP53*, ongoing UV exposure will facilitate expansion of the clone as well as inhibit immune-mediated surveillance. Chronic UV exposure results in development and expansion of additional clones that evolve and compete with normal cells and each other in a field of actinically damaged skin. Accumulation of additional mutations over time and ongoing UV exposure will allow progression of a microscopic subclone into a visible actinic keratosis and eventually invasive squamous cell carcinoma.

Based on these data, a mechanistic model of field cancer emerges, in which initial exposure to UV radiation causes sporadic somatic mutations and subsequent UV exposure induces clonal expansion of these mutants and inhibits immune surveillance of these malignant precursors ( Figure 3). Competition between mutant clones and normal cells facilitates large patches of mutated cells within an actinically damaged field. Over time, the accumulation of additional mutations will allow progression to visible lesions of AK and eventually SCC. The existence of multiple clones harboring distinct and potentially carcinogenic mutations in normal skin was recently confirmed: deep sequencing of aged eyelid skin identified somatic mutations in 18–32% of epidermal cells, and the total mutation burden was comparable to many adult solid tumors ^43^. These clonal proliferations are the earliest detectable precursors of SCC and represent targets for therapeutic intervention to prevent subsequent skin cancer. What remains unknown, however, is whether specific variables such as the frequency, size, total mutation burden, or specific mutation profile of these clones determine the magnitude of SCC risk.

## Management of field cancerization

The first step in the management of the patient with multiple SCCs and field cancerization is rigorous sun protection. Because UV radiation is both a tumor initiator and a tumor promoter for skin cancer and because animal models demonstrate reduced precursor lesions when UV exposure is interrupted ^42^, there is a strong incentive to limit exposure even in patients with established actinic damage. The most compelling data to support sun protection come from randomized trials performed in Australia, a region of intense environmental UV radiation and a susceptible population with the greatest incidence of SCC worldwide. In 1993, Thompson and colleagues randomly assigned 588 patients with established AK to daily use of either sun protection factor (SPF) 17 broad-spectrum sunblock (UVA and UVB protection) or placebo cream. Patients who received sunblock had a significant reduction in new AK, and this reduction was proportional to the total amount of sunblock used ^44^. Of note, the use of a placebo sunblock would likely be considered unethical in 2018, but the benefits of sunblock were not yet established at the time of the trial. A second trial randomly assigned 1,621 unselected participants to one of four treatments: daily use of SPF 15 broad-spectrum sunblock, discretionary use of sunblock, daily sunblock plus oral beta-carotene, and oral beta-carotene alone with discretionary use of sunblock. Over a follow-up period of 4 years, daily sunblock use caused a significant 39% reduction in new SCC; the use of beta-carotene had no effect on skin cancer development ^45^. Follow-up of these participants for an additional 8 years revealed a persistent 41% reduction in new SCC in the former daily sunblock group, even when returning to discretionary sunblock use after completion of the original trial ^46^. Finally, there is an important role for sun protection in immunosuppressed organ transplant recipients as well. In a 24-month trial of organ transplant recipients at high risk of SCC, patients provided with SPF 50 broad-spectrum sunblock developed fewer SCCs (eight in the control group versus zero in the sunblock group) and exhibited a significant regression of existing AK ^47^. Cumulatively, this suggests that rigorous sun protection will have significant and long-lasting benefits in both prevention and management of field cancerization.

Apart from sun protection, the most widely used field-directed treatments are topical medications such as the pyrimidine analog chemotherapeutic 5FU. For many years, the observation that SCC precursors can be effectively treated with topical therapy provided the rationale for field treatment to decrease the incidence of SCC in high-risk patients. There is strong evidence to support the use of 5FU and other topical agents, such as the immune response modifier imiquimod and the non-steroidal anti-inflammatory drug diclofenac in the treatment of AK. Clinical evidence for these agents in AK treatment is extensive and beyond the scope of this review ^48^. The hypothesis that treatment of precursor lesions within a cancerized field can reduce subsequent SCC development is supported by several lines of indirect evidence and, more recently, a clinical trial that documented clear efficacy of 5FU in SCC prevention. First, there is a proven benefit of field therapy over lesion-directed therapy for sustained clearance of AK within a defined field. One randomized trial found that although 5FU, imiquimod, or lesion-directed destructive therapy all had good immediate efficacy in AK clearance (68–96%), only field therapy with 5FU or imiquimod produced any significant clearance at 1 year (33% and 73%, respectively, versus 4% for lesion-directed therapy) ^49^. Second, long-term follow-up of patients randomly assigned to 5FU field therapy versus placebo demonstrated that the benefits of sustained lesion clearance are significant for over 3 years following a single treatment course, suggesting that topical field therapy produces a long-lasting remission in SCC precursors ^50^. Third, a recent randomized, double-blind clinical trial in 932 patients with a history of skin cancer (39% of whom had prior SCC) identified a 75% reduction in incident SCC in the first year following a single course of topical 5FU treatment (five patients with SCC in the 5FU group versus 20 patients in the placebo group) ^51^. This study is the first high-quality, prospective trial to demonstrate a significant reduction in SCC with field therapy and provides strong support for the concept of field-directed therapy in high-risk patients. Finally, preclinical and early clinical data suggest that topical 5FU can be combined with topical immunotherapy to enhance field-directed prevention of SCC. Topical calcipotriol, a vitamin D derivative shown to enhance cutaneous T-cell activation via the production of thymic stromal lymphopoietin (TSLP), can reduce the development of SCC in animal models and was shown to enhance the efficacy of AK clearance with 5FU in a preliminary human trial ^52^.

Another topical field therapy has recently emerged in the form of ingenol mebutate, a cyclic diterpene ester derived from the sap of the *Euphorbia peplus* plant, which itself is a known irritant and established botanical therapeutic ^53^. Ingenol mebutate has direct cytotoxic effects on keratinocytes and induces a local inflammatory milieu via effects on the microvasculature and endothelial cells. Unlike other topical therapies, a short, 2- to 3-day treatment with ingenol mebutate has demonstrated efficacy in field treatment of AK, which may improve patient compliance ^54, 55^. Via elimination of SCC precursors such as *TP53*-mutated clones in the epidermis, ingenol mebutate can prevent or reduce the development of UV-induced lesions in an animal model of SCC ^56^. Similarly, ingenol mebutate therapy in humans has been shown to decrease not only visible AK but also subclinical precursor lesions detected by optical coherence tomography and histopathologic analysis ^27^. Although it appears likely that various topical agents such as ingenol mebutate and imiquimod can decrease the incidence of subsequent SCC in patients with field cancer, direct evidence of this effect is currently available only for 5FU ^51^.

An established option for field-directed treatment is office-based PDT. PDT is based on photo-activation of protoporphyrin IX in keratinocytes to generate reactive oxygen species and cytotoxicity. A prodrug (aminolevulinic acid or methyl aminolevulinate) is topically applied to the skin 1–18 hours prior to visible-spectrum light treatment, and because metabolic conversion of the prodrug to protoporphyrin IX is enhanced in malignant and pre-malignant cells, this treatment is relatively selective for SCC precursors in the skin ^57^. Analogous to the topical therapies presented above, PDT is an effective field treatment for AK and is superior to lesion-directed destructive therapy ^58^. Like other field treatments, PDT can significantly reduce the size and number of *TP53*-mutated clonal precursors of SCC in human skin ^59^ and has been demonstrated to decrease UV-induced tumor development in animal models ^60^. Because PDT (and presumably other current field therapy options) does not completely eliminate mutated precursors from the skin, repeated treatments appear to be required to continually suppress the development of SCC. One observational study of 12 organ transplant recipients with high-risk field cancer treated with cyclic PDT every 4–8 weeks reported 79% and 95% reductions in incident SCC after 1 and 2 years, respectively, compared with the year prior to cyclic PDT ^61^. In contrast, a prospective trial of 40 patients who received a single course of PDT found no significant decrease in SCC incidence after 2 years ^62^. The optimal interval for cyclic therapy may be better defined by an ongoing trial investigating cyclic PDT every 6 months in organ transplant recipients at elevated risk for skin cancer; interim results of the trial have not yet detected any incident SCC ^63^. If repeated treatments are required, one promising new alternative could be daylight PDT, in which photo-activation of protoporphyrin IX is achieved by natural daylight as opposed to more intense (and often more painful) artificial light sources. Although daylight PDT appears promising for the treatment of AK ^64^, its effect on SCC prevention has not been investigated.

For patients with severe field damage at highest risk for subsequent SCC, systemic treatment may dramatically inhibit new tumor development. Acitretin is a systemic retinoid known to suppress proliferation and promote differentiation of keratinocytes and has been shown to induce regression of SCC-like tumors in animal models ^65^. Compelling evidence to support acitretin as field therapy comes from a prospective trial of 44 renal transplant recipients randomly assigned to either acitretin 30 mg daily or placebo for 6 months. Incident SCC was reduced by 88% in patients during acitretin treatment, although the rate of SCC development increased to the same as the control group in the 6 months following discontinuation of therapy ^66^. Similar to PDT or topical therapy, field treatment with acitretin suppresses the development of SCC but does not completely eliminate clonal precursors of SCC and thus therapy must be continued for a prolonged period of time. Systemic side effects from acitretin can be significant but are predictable and often dose-dependent, including xerosis, mucositis, myalgias and arthralgias, hyperlipidemia, and potential hepatotoxicity. Acitretin, like all systemic retinoids, is an established teratogen and should be avoided in women of child-bearing potential because of its long half-life. Isotretinoin has a significantly shorter half-life and may be an alternative for younger women, although it is rare for this population to require systemic field therapy ^67^. It should also be noted that the beneficial effects of systemic therapy are less pronounced in patients at lower risk for SCC and this therapy should be reserved for patients with few other options ^68^.

At least two other approaches for systemic field therapy may also be considered. First, nicotinamide (also known as niacinamide, the amide form of vitamin B _3_) has recently been shown to have modest preventive effects on UV-induced skin lesions, presumably by promoting effective DNA repair and reducing the immunosuppressive effects of UV exposure ^69^. In a prospective trial of 386 patients with prior skin cancer, those randomly assigned to oral nicotinamide 500 mg twice daily had a 23% reduction in new skin cancers (30% reduction in SCC) during 12 months of therapy. When patients were monitored for 6 months after cessation of nicotinamide therapy, there was a non-significant trend toward increased SCC incidence during this period ^70^. Although nicotinamide produced only a modest reduction in SCC incidence, the lack of significant side effects associated with this agent makes it a potentially useful option, either alone or in combination with other field therapies. Second, capecitabine, an oral prodrug of 5FU that is US Food and Drug Administration-approved for the treatment of colorectal, gastric, and breast cancer, may be an effective prophylactic measure for patients with recalcitrant field cancerization. In one uncontrolled observational study, cyclic treatment with capecitabine produced a 68% reduction in incident SCC ^71^. Because of the significant side effect profile of this systemic chemotherapy, treatment of cutaneous field cancerization with capecitabine is usually performed in conjunction with a medical oncologist.

## Approach to the patient with field cancerization

**Figure 4.**
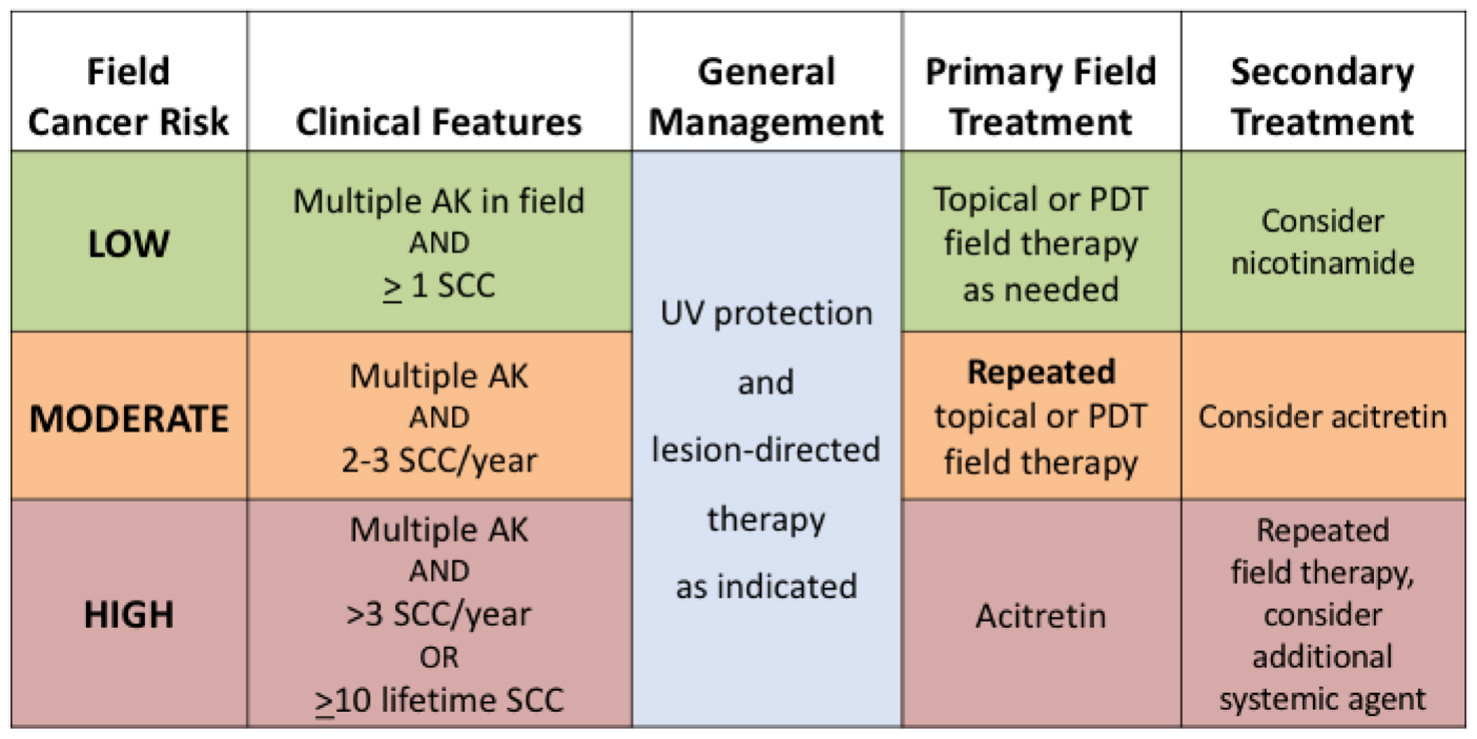
Approach to the patient with field cancerization. Field cancer treatment is based on risk of subsequent squamous cell carcinoma (SCC), as determined by clinical factors. All patients require ultraviolet (UV) protection and lesion-directed therapy as indicated. AK, actinic keratosis; PDT, photodynamic therapy.

In order to appropriately manage patients with cutaneous field cancer, it is necessary to define this term. A practical working definition of field cancerization requires three features: a defined region of skin, multiple AKs within that region, and at least one prior SCC. Although patients with severe actinic damage and multiple AKs without prior SCC may certainly require treatment for their AKs ^72^, those patients are not considered here. The type, frequency, and intensity of field treatment for an individual patient depend on the severity of field cancerization and the risk of subsequent SCC ( Figure 4). All patients with evidence of field cancerization should be counseled on rigorous sun protection and have appropriate lesion-directed therapy of SCC and hypertrophic AK as indicated. Patients with multiple AKs and few sporadic SCCs are at relatively low risk of subsequent SCC and can be managed with occasional field therapy according to patient or physician preference. This may include topical 5FU, imiquimod, or ingenol mebutate, or PDT. Patients with a consistent pattern of two or three SCCs per year are at increased risk of continual SCC development and will require repeated cycles of field therapy at some interval ranging from annually to every few months. As noted previously, current field therapies suppress, rather than completely eliminate, the precursors of SCC; repeated treatments are often required for ongoing suppression. A combination of simultaneous field therapy and lesion-directed therapy in these patients may also improve local control ^73^. Those not responding to PDT or topical therapy may benefit from systemic acitretin. Patients with more than three or four SCCs per year or more than 10 lifetime SCCs, as noted previously, are at high risk of both subsequent SCC and metastatic or fatal SCC and require aggressive field therapy. Initial therapy in these patients is often systemic acitretin, which may be combined with repeated topical therapy or PDT. Combination treatments may have synergistic efficacy, as reported in one recent series of combined lesion-directed destruction, topical 5FU, and PDT ^74^. Combination systemic therapy with nicotinamide and acitretin is generally accepted as safe, but concurrent use of acitretin and capecitabine has not been studied and should be used with caution. Finally, organ transplant recipients require additional focus on management of field cancer, as the chronic use of immunosuppressant medications places these patients at exceptionally high risk of multiple and biologically aggressive SCCs ^75^. Organ transplant recipients often require more frequent monitoring, earlier initiation of multi-modal field therapy, and potentially modification of their immunosuppression regimen in collaboration with their transplant team.

## Conclusions and future directions

Significant progress has been made in understanding the pathogenesis and optimal management of field cancerization, yet several questions remain. It is clear that patients with a large burden of cutaneous SCC have a chronic disease with an increased risk of systemic involvement and early death; treating each lesion as an isolated neoplasm does not adequately address this disease. A systemic and systematic approach to field therapy is required for these patients. It is also clear that SCC evolves from microscopic precursors harboring carcinogenic mutations in grossly normal skin and that a significant fraction of epidermal cells may harbor these mutations in an actinically damaged field. It remains unclear, however, whether the number, size, total mutation burden, or specific mutation profile of these precursors is the critical determinant of progression to SCC. With additional data from genetic sequencing of normal but sun-exposed skin, it may be possible to more accurately determine the risk of SCC development, both for a single precursor lesion and for a cancerized field as a whole. Similarly, nearly all patients with more than 10 SCCs presented initially with a single lesion, yet current methods cannot prospectively determine subsequent field cancer risk. Additional study is required to identify those patients who would benefit most from field treatment at an early point in their field disease progression. Although there is now high-quality evidence to show that field treatment with topical 5FU can reduce the incidence of SCC for at least 1 year, additional clinical trials are needed to support the use of other therapies and the optimal interval of repeated field therapy for high-risk patients. Finally, anti-tumor immunity has been shown to be a critical factor in host defense against cancer, but the role of the immune response in the development and progression of precursor lesions has only been suggested. Future field treatments may more effectively harness the immune system to achieve a durable suppression of SCC development in a cancerized field.
